# Elucidation and analyses of the regulatory networks of upland and lowland ecotypes of switchgrass in response to drought and salt stresses

**DOI:** 10.1371/journal.pone.0204426

**Published:** 2018-09-24

**Authors:** Chunman Zuo, Yuhong Tang, Hao Fu, Yiming Liu, Xunzhong Zhang, Bingyu Zhao, Ying Xu

**Affiliations:** 1 College of Computer Science and Technology, Jilin University, Changchun, China; 2 Computational Systems Biology Lab, Department of Biochemistry and Molecular Biology and Institute of Bioinformatics, University of Georgia, Athens, GA, United States of America; 3 Noble Research Institute, LLC., Ardmore, OK, United States of America; 4 North Automatic Control Technology Institute, Taiyuan, China; 5 Department of Crop and Soil Environmental Science, Virginia Polytechnic Institute and State University, Blacksburg, Virginia, United States of America; 6 Department of Horticulture, Virginia Polytechnic Institute and State University, Blacksburg, Virginia, United States of America; Jawaharlal Nehru University, INDIA

## Abstract

Switchgrass is an important bioenergy crop typically grown in marginal lands, where the plants must often deal with abiotic stresses such as drought and salt. Alamo is known to be more tolerant to both stress types than Dacotah, two ecotypes of switchgrass. Understanding of their stress response and adaptation programs can have important implications to engineering more stress tolerant plants. We present here a computational study by analyzing time-course transcriptomic data of the two ecotypes to elucidate and compare their regulatory systems in response to drought and salt stresses. A total of 1,693 genes (target genes or TGs) are found to be differentially expressed and possibly regulated by 143 transcription factors (TFs) in response to drought stress together in the two ecotypes. Similarly, 1,535 TGs regulated by 110 TFs are identified to be involved in response to salt stress. Two regulatory networks are constructed to predict their regulatory relationships. In addition, a time-dependent hidden Markov model is derived for each ecotype responding to each stress type, to provide a dynamic view of how each regulatory network changes its behavior over time. A few new insights about the response mechanisms are predicted from the regulatory networks and the time-dependent models. Comparative analyses between the network models of the two ecotypes reveal key commonalities and main differences between the two regulatory systems. Overall, our results provide new information about the complex regulatory mechanisms of switchgrass responding to drought and salt stresses.

## Introduction

Switchgrass (*Panicum virgatum L*.) is a warm-season C4 grass native to North America, and is considered a major biofuel crop for cellulosic ethanol production. Because of its strong adaptability and rapid growth [1–4], switchgrass is primarily grown in marginal lands [5], which tend to be affected by abiotic stress, such as drought and salt stresses [6, 7]. Recent studies suggest that these two are the major factors that may limit the biofuel production [8–10]. Hence, it represents an important task to understand how plants adapt to these stresses and how to possibly engineer switchgrass to make the plant more tolerant to drought and salt stresses.

Previous studies have reported that lowland ecotypes tend to be more tolerant to drought and salt than the upland ecotypes determined based on specific physiological, morphological, or metabolic parameters [10, 11]. Specifically, (i) lowland ecotypes tend to have higher levels of the following under drought stress: photosynthetic rates (*P_n_*), transpiration rates (*T_r_*), stomatal conductance (*g_s_*), intercellular *CO*_2_ (*C_i_*) level and relative water content (RWC) in leaves, and lower electrolyte leakage (EL), in comparison with the upland ecotypes; and (ii) they generally have lower levels of salt stress-induced cell-membrane damages due to their higher levels of *P_n_*, *g_s_*, *T_r_*, leaf photochemical efficiency and chlorophyll content (Chl content) and lower EL content. As of now, no genetic-based research has been published regarding detailed understanding of what specifically make one ecotype more tolerant to these stresses than the other.

Here we present a computational study of public time-course gene-expression data of Alamo (a lowland ecotype of switchgrass) and Dacotah (an upland ecotype) treated with drought and salt stresses. The goals are (1) identification of key biological processes that respond to each of the two stresses; (2) inference of the functional relationships among the identified processes, hence providing a systems level understanding about the response program to each stress; and (3) comparison of commonalities and differences in the response processes between the two ecotypes, aiming to gain improved understanding of what may have made one ecotype more stress tolerant than the other.

A key in addressing these questions, particularly (2) and (3) lies in identification of the main transcription regulators (TFs) of the response programs to the two stress types. To accomplish this, we have identified all differentially expressed TFs in stress-treated vs. untreated switchgrass samples. In addition, we have also identified differentially expressed genes (TGs) known to play key roles in stress response and adaptation, such as biosynthesis of osmoprotectants; control of the water flux; adjustment of the cellular osmolarity [12, 13]; influx and efflux through plasma membrane and vacuole relevant to intracellular ion homeostasis [14]; maintaining redox homeostasis [15]; reprogramming of plant primary metabolisms; and alteration of the cellular architecture for more efficient energy supply [16]. Based on the identified genes in these categories, we have predicted a regulatory network for the response system to each stress, consisting of key TFs and predicted regulatory relationships between the TFs and functional TGs involved in these metabolic processes, which is most consistent with the time-course data collected from the public domain.

Overall, two regulatory networks are predicted, one with 143 TFs, 1,693 stress-response TGs and 11,784 TF-TG regulatory relationships for drought stress, and the other having 110 TFs, 1,535 stress response TGs and 8,411 TF-TG relationships for salt stress. Among the predicted TF-TG relationships, 46.1% and 43.5% in the drought and salt response programs are validated against two independent data sources, respectively. Comparative analyses revealed that Alamo has different stress response systems from those in Dacotah.

## Materials and methods

### Data

Two ecotypes of switchgrass: Dacotah and Alamo are used in the study. The transcriptomic data of drought and salt-stressed Dacotah and Alamo used in this study are retrieved from the Sequence Read Archive (SRA) [17] with recorder numbers PRJNA323435 and PRJNA323349, respectively. Specifically, plants of each ecotype to be stress-treated, along with the controls, are grown in separate pots in a greenhouse [11].

Once the plants reach the E5 development stage after two months [18], they are exposed to one of two stress treatments: drought or salt stress, for 30 days, separately. Leaf tissue samples from the same plants of the two ecotypes are taken at six (0d, 6d, 12d, 18d, 24d, 30d) and eight (0h, 12h, 24h, 48h, 6d, 12d, 18d, 24d) time points after treatment of drought and salt stresses, respectively, along with the matching leaf samples collected from the untreated plants, for RNA-Seq analyses. To minimize the leaf age effect, we have four tillers from the original single tiller transplanted for each pot and at each sampling time, and cut the same section of the leaf blade (~100 mg) of the top 2^nd^ and 3^rd^ fully developed leaves from tillers with the same size. For each time point, three biological replicates are made, giving rise to a total of 72 and 96 samples for the two ecotypes, respectively. It is noteworthy that 3 pots are used for each treatment along with 3 controls pots, with each treatment having its own control pot. All plants for each ecotype are the clonal replicates of a single mother plant. These two data sets are of high-quality as reflected by that the biological replicates for each sample have strong correlations among themselves, with Pearson correlation coefficient (PCC) > 0.9.

The switchgrass genome version 4 (Pvir_v4) and annotation are downloaded from JGI [19]. Protein sequences encoded in three model species: Arabidopsis, maize and rice are also downloaded from JGI and used in this study. 4,134 TFs of switchgrass and GO-based functional annotation of switchgrass genes are downloaded from PlantTFDB [20]. The predicted TF-TG interactions in Arabidopsis, maize and rice are collected from the literature [21–26], respectively.

### RNA-Seq data processing

The downloaded files of gene-expression data for the 72 and 96 samples are converted into the fastq format using fastq-dump command in the SRA Toolkit (version 2.8.2–1) [27]. The following data-processing steps: adapter-trimming, quality-trimming, filtering and contaminant-filtering, are applied to each fastq file using the BBDuk tools (version 35.82) [28], a JGI pipeline [29] for filtering and trimming RNA reads containing adapters and contaminants. Hisat2 (version 2.1.0) [30, 31] is used, with default parameters, to map RNA reads onto the Pvir_v4 genome. Gene-level raw RNA-read counts are summarized using FeatureCounts (version 1.5.0) [32, 33] from the aligned sam files. Two and four samples with less than 70% total alignments to the genome [34] are removed from the two collections of samples, leaving 70 and 92 samples for drought and salt stresses, respectively (S1 Table).

We have estimated the scaling factors between samples from two ecotypes for each stress by using the Trimmed Mean of M-values (TMM) in the edgeR package (version 3.20.9) [35, 36]. The count per million (CPM) is computed using the ‘cpm’ function provided in the same package to estimate the gene-expression levels. Only genes with expression levels higher than 2 CPM in more than 90% of the samples for each stress type are kept. At the end, 20,581 and 20,836 genes for drought and salt stresses are used for further analyses, respectively.

### Statistical analysis

The raw RNA-read counts are put into the edgeR package [35, 36] and used to determine the differentially expressed genes (DEGs). For each treated sample, a fold-change value (after log transformation) and the associated p-value are calculated against the matching untreated samples. Each gene with absolute fold change > 2 and p-value < 0.05 is considered as a DEG. A gene in an ecotype is considered as *stress affected* if it is a DEG at two time-points or more among the stress treated samples of the ecotype. For each stress and each ecotype, 1,500 stress affected genes with the lowest p-values are kept to construct a gene regulatory network (GRN). Overall, 2,441 and 2,429 stress affected genes for drought and salt stresses are kept for the two ecotypes, respectively, each referred as *stress-responding* gene, of which 147 and 125 are TFs.

### Construction of genetic regulatory network

The initial GRN is constructed based on co-expression information measured using the Pearson correlation coefficient. Specifically, a pair of TF and TG with PCC > 0.70 is predicted to have a regulatory relationship. Then, this network is put into the Network Component Analysis (NCA) algorithm (version 2.3) [37, 38] to calculate differential expression profiles of the stress-regulated TFs, where NCA is a method for reconstructing TF activities (TFAs) and estimating control strengths (CSs) through estimating the partial and qualitative network connectivity information based on gene-expression data. Specifically, an NCA model is described as:

[X]=[A][P]

where *X* is a matrix of gene expression values, with rows for genes and columns for samples; *A* is a binary matrix of (predicted) regulatory relationships between TFs (columns) and TG (rows), and *P* is a TFA matrix with columns for samples and rows for TFs; and *A*_*i*,*j*_ is 1 if and only if there is a regulatory relationship between TF *j* and gene *i*; otherwise 0. A desired decomposition of *X* to *A* and *P* is achieved by minimizing the following objective function:

$$\min{\left|\left|\left[X]-[P][A\right]\right|\right|}^{2},$$

under the following constraints:

1. [*A*] has full column-rank;
2. when a node in the regulatory layer is removed along with all the output nodes connected to it, the connectivity matrix for the resulting network must have full column-rank; and
3. [*P*] has full row-rank.

Each TF-TG interaction predicted by NCA and its PCC ≥ 0.75 is regarded as the final prediction for a regulatory relationship.

### Validation of predicted TF-TG interactions

It has been widely observed that TF-TG relationships tend to be conserved across related species [39–42]. Hence, we have used known TF-TG relationships (see Data) in related organisms to validate our TF-TG predictions. InParanoid (version 4.1) [43, 44] is applied to generate orthologous groups between switchgrass and Arabidopsis, maize as well as rice, respectively. We consider a predicted TF-TG interaction as validated if it has orthologous pairs of TF-TG interactions in the above six databases.

### Validation of predicted TGs for each TF

To quantify functional relevance of the predicted TGs for each TF, we have calculated GO semantic similarity scores of the GO terms between each pair of the co-regulated TGs, using the GOSemSim package (version 2.6.0) [45, 46]. Here, we considered only the GO biological processes. To evaluate the statistical significance of the derived functional similarity among the TGs regulated by the same TF, we have randomly selected the same number of genes from the background gene set, namely a set of all the 25,971 switchgrass genes with GO annotations, and calculated their functional similarities. Wilcoxon rank-sum test is used to assess whether the calculated functional similarity scores among the TGs are significantly higher than those among randomly selected genes.

### Dynamic regulatory maps

The Dynamic Regulatory Event Miner (DREM) (version 2.0.3) [47, 48] is used to reconstruct the relevant regulatory relationships in each ecotype under each stress. Specifically, DREM takes time-course fold-changes of TGs and predicted TF-TG interactions as inputs, and produces a dynamic regulatory map consisting of multiple time-dependent chains of events. Each chain consists of a collection of co-expressed genes across all the time points, measured using fold changes in expression levels with respect to time 0 as the reference, where there are TFs that co-regulate genes at time t and time (t+1), across all time points. An important property of the modeling technique is that it allows the formation of bifurcation events at each time point if co-expressed genes up till the current point diverge into two distinct groups of co-expressed genes from the current time point on within each group but not between the two groups. Each bifurcation event is associated with two sets of TFs that each regulate a subset of all the genes, where the TF split cutoff score is 0.001.

### GO enrichment analysis

GO term enrichment is conducted over a given gene set using the topGO R package (version 2.30.1) [49, 50], where all annotated genes in switchgrass is used as the background. A GO function is considered enriched if the p-value < 0.01.

The protocol for the entire computational analysis pipeline, used in this study, has been published in protocols.io with the following DOI: dx.doi.org/10.17504/protocols.io.saxeafn.

## Results

Considering that only one switchgrass genome, namely that of Alamo, is published [19], we have mapped all the RNA-Seq reads of both ecotypes to the same genome when estimating gene-expression levels in each ecotype under each stress.

### DEGs responding to drought and salt stresses

**Fig 1:**
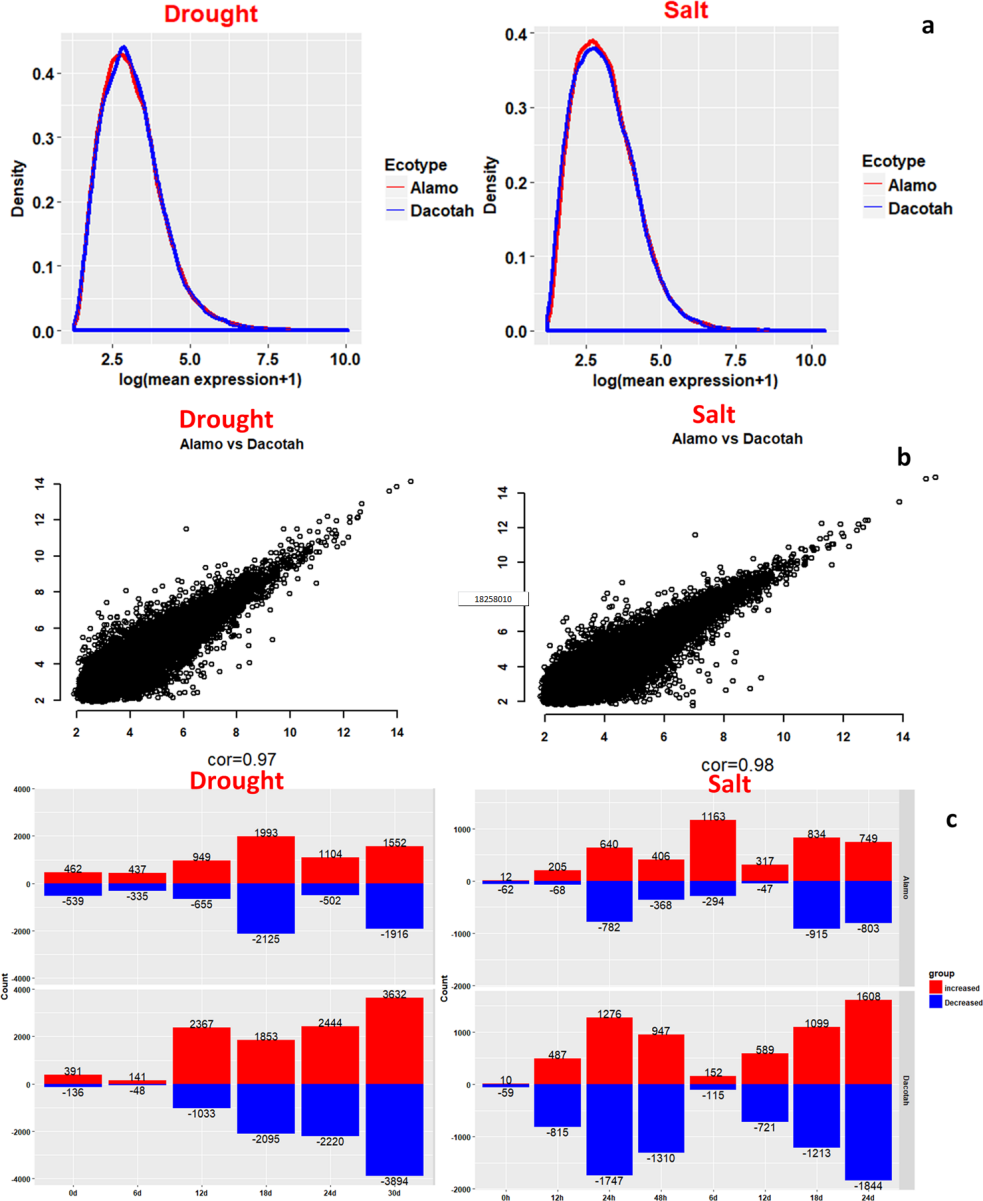
Characterization of gene-expression profiles of each ecotype under drought and salt stresses. (a) The distribution of log (mean CPM+1) for each ecotype. (b) The scatterplots for PCCs between expressions of each gene in the two ecotypes averaged over all relevant samples under drought and salt stress, respectively. (c) The number of DEGs for each ecotype at each time point for each stress. The number of up- and down-regulated genes, marked as red and blue, respectively, for each combination of ecotypes and stresses.

Comparison between the two ecotypes under each stress type revealed: they have similar gene expression profiles at the global scale, as shown by their similar expression-level distributions (Fig 1A) and high statistical correlations between gene expressions of the two ecotypes under the same stress type (Fig 1B).

Our differential gene-expression analyses have revealed that there are significant differences between the two ecotypes in terms of their responses to the two types of stresses under study (Fig 1C, S1 File). First, more DEGs are detected under the drought stress than the salt stress for both ecotypes, suggesting that drought has larger impacts on the plant than salt, and requires more diverse cellular responses, which is also observed in *Brassica napu*s [51]. For each stress, there are more DEGs in Dacotah than in Alamo almost at every time point (see Methods). We have also noted that the numbers of up- vs. down-regulated genes are approximately the same for both ecotypes under each of the two stress types, which is consistent with previous studies regarding abiotic stress response in Arabidopsis [52].

Interestingly, there is one time-point (6d for salt and 18d for drought) (see Methods), where the number of up-regulated genes in Alamo is higher than Dacotah, which is different from all the other time points. A key function enriched by the up-regulated genes at this point for salt stress is photosystem II assembly and stabilization, a known adaptive mechanism for protecting plants from permanent damages [53]. Previous studies have observed that Alamo has higher photosynthetic rates than Dacotah [10]. Hence, we posit that this energy-related mechanism may play a key role in protecting Alamo from damages.

Overall, 676 distinct TFs are found to be differentially expressed in total across the two ecotypes under two stresses (Panel a in S1 Fig), specifically, 207, 266, 361 and 531 for Alamo and Dacotah under drought and salt, respectively. We not e that most of these TFs are in the families of bHLH, bZIP, NAC, G2-like, MYB_related and ERF (Panel b in S1 Fig), which are all known to play important roles in abiotic stress response [54, 55]. Of these TFs, 87 are found in all four combinations of the two ecotypes under two stresses; and 64 additional (distinct) ones are in Alamo and 233 ones in Dacotah. See S2 File for details.

### GRNs for the two stress types

We have constructed a GRN from the identified stress-responding genes (see Methods) for each stress type, respectively. The network consists of a set of stress-responding TFs and TGs regulated by such TFs, namely TFAs as defined earlier. Overall, 100 and 95 TFs are identified in Alamo and Dacotah under drought, respectively; and 84 and 77 TFs are identified in Alamo and Dacotah under salt, respectively.

We have then conducted co-expression analyses between the TFs and the TGs identified for each stress type, where a pair of TF and TG is considered as having a regulatory relationship if their PCC > 0.70 [25, 56]. This results in the initial prediction of the regulatory relationships in the response system to the drought and salt stresses, respectively.

Overall, 37,896 and 16,350 regulatory interactions are predicted between 147 TFs and 2,262 TGs under drought and between 125 TFs and 1,893 TGs under salt (Table 1), respectively. These regulatory relationships and associated expression data are then fed into the NCA algorithm (see Methods), after 4 and 15 TFs being removed with the number of TGs less than two or co-regulating with other TFs, which give rise to the TFA profiles for the 143 and 110 TFs as the response systems to drought and salt, respectively, as detailed in S2 and S3 Figs. All the 143 and 110 TFs exhibit distinct expressions for two ecotypes across each of the six and eight time-points under the drought and salt stress, respectively, indicating different cellular responses at different time points for the two ecotypes under the same stress.

**Table 1:** Summary of GRNs of switchgrass in response to drought and salt stresses, respectively.

Stress	Processing	#TFs	#Targets	#Interactions	#Positive interactions	#Negative interactions
Drought	PCC correlation >0.70	147	2,262	37,896	33,548	4,348
	NCA prediction	143	1,844	21,239	18,149	3,090
	PCC correlation ≥ 0.75	143	1,693	11,784	10,806	978
Salt	PCC correlation >0.70	125	1,893	16,350	14,278	2,072
	NCA prediction	110	1,831	14,956	12,906	2,050
	PCC correlation ≥ 0.75	110	1,535	8,411	7,577	834

Positive and negative interactions represent those with PCCs > 0 and < 0, respectively.

**Fig 2:**
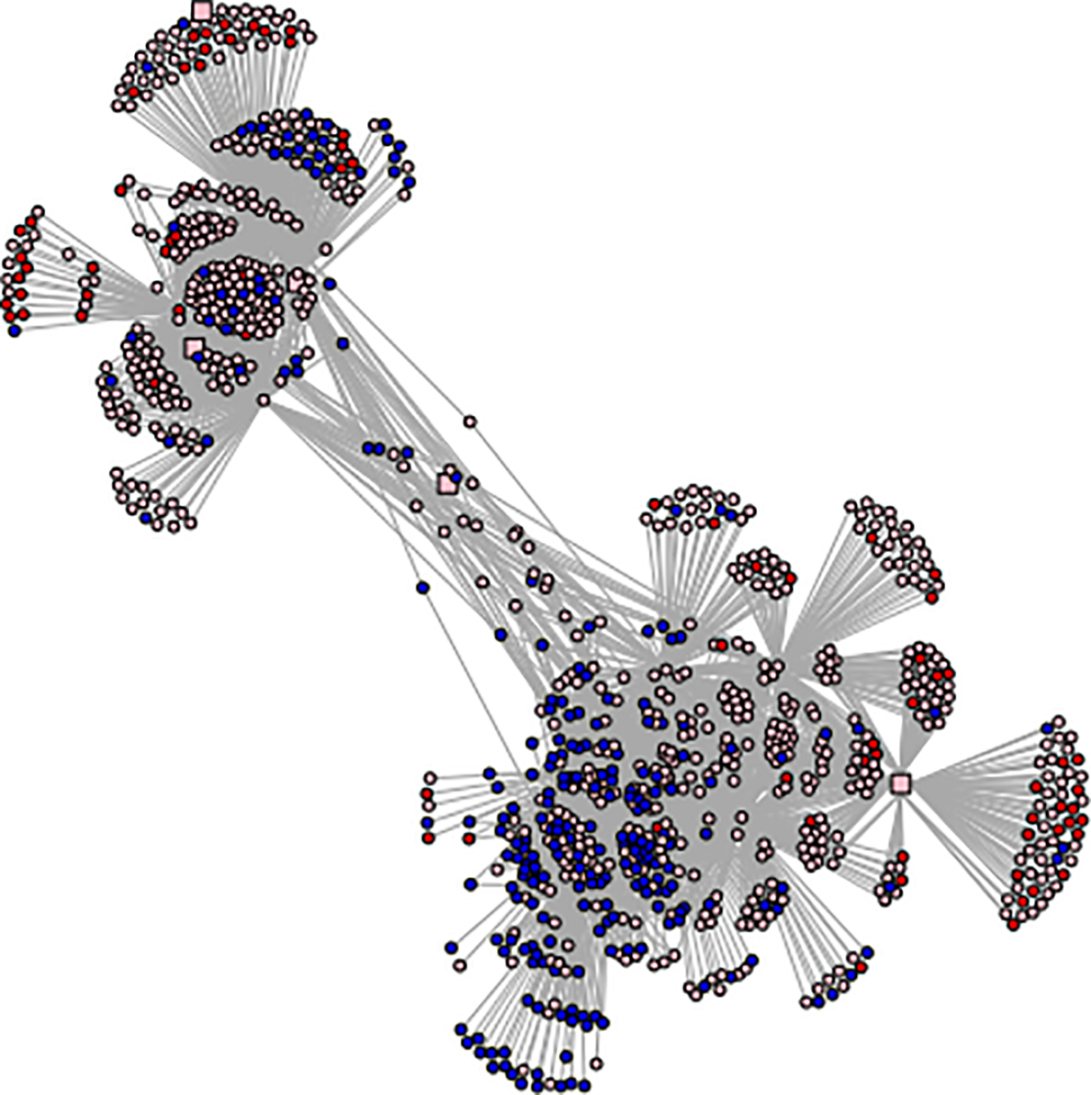
The predicted GRNs (partial) for 15 TFs with the highest numbers of TGs of switchgrass under drought stress. TFs and TGs are represented as squares and circles, respectively. Genes regarded as stress affected DEGs for both ecotypes, only in Alamo and Dacotah are marked in pink, red and blue, respectively.

We have next conducted functional analyses of the predicted regulatory relationships. First, we have applied a higher PCC cutoff 0.75 (S4 Fig) to the predicted regulatory relationships so we can focus on the most reliable predictions. This gives rise to 11,784 and 8,411 high-confident TF-TG interactions (S3 File) in the combined lists of the two ecotypes for drought and salt stresses, respectively. Table 1 summarizes these positive (activation) and negative (repression) regulatory relationships. From the structure of partial drought and salt networks (Fig 2, S5 Fig) generated by using the igraph package (version 1.2.1) in R [57, 58], we note that some TF-TGs primarily occur in the GRN only for one ecotype while others in both ecotypes.

### Validation of predicted regulatory interactions

We have validated the predicted TF-TG relationships using two independent data sources: (1) TF-TG relationships in Arabidopsis, maize and rice as such relationships tend to be conserved across related plants, and (2) TGs regulated by the same TF should have highly related or similar functions.

First, we have compared the predicted TF-TG relationships with known TF-TG pairs in Arabidopsis, maize and rice (see Methods). Specifically, we have mapped 503 and 408 predicted regulatory pairs of switchgrass to the orthologous TF-TG pairs in Arabidopsis, maize or rice for drought and salt network, respectively, hence considered them as validated as detailed in Table 2 and S4 File.

**Table 2:** Validation of the predicted TF-TG interactions by orthologous pairs in Arabidopsis, rice and maize, respectively.

Species	Drought	Salt	Reference
Arabidopsis	66 (38)	77 (37)	[24]
	182 (15)	45 (14)	[21]
	4 (3)	6 (4)	[25]
	202 (48)	261 (47)	[26]
Rice	0 (0)	4 (1)	[23]
Maize	101 (34)	72 (29)	[22]
Total	503 (79)	408 (70)	N/A

The number inside each pair of parentheses represents the number of TFs involved in the corresponding interactions.

We have then checked the TGs regulated by the same TFs in terms of their functional relevance measured using the GO Biological Processes (see Methods). Among the 11,784 predicted TF-TG interactions, 5,212 interactions between 139 TFs and 815 TGs in the drought response GRNs have functional relevance (using cutoff 0.3, Panel a in S6 Fig), based on an saturation analysis of functional similarities [59] among TGs regulated by the TF. Similarly, among the 8,411 TF-TG interactions, 3,425 interactions between 97 TFs and 696 TGs in the salt GRNs have functional relevance. All these are considered as validated based on functional relevance information. This clearly provides strong support to our predicted regulatory networks. The detailed information is summarized in S5 File.

Overall, 5,434 (46.1%) and 3,657 (43.5%) TF-TG interactions in the predicted GRNs in response to drought and salt, respectively, are considered as validated by the above two approaches. Although false positive interactions may inevitably be present in our prediction, our benchmark analysis (Panel b in S6 Fig) demonstrates that the average GO annotation similarities of drought and salt network are significantly higher than that of a random gene interaction network (p-value 2.2*e*^−16^).

### Functional analyses of the predicted stress-response networks

**Fig 3:**
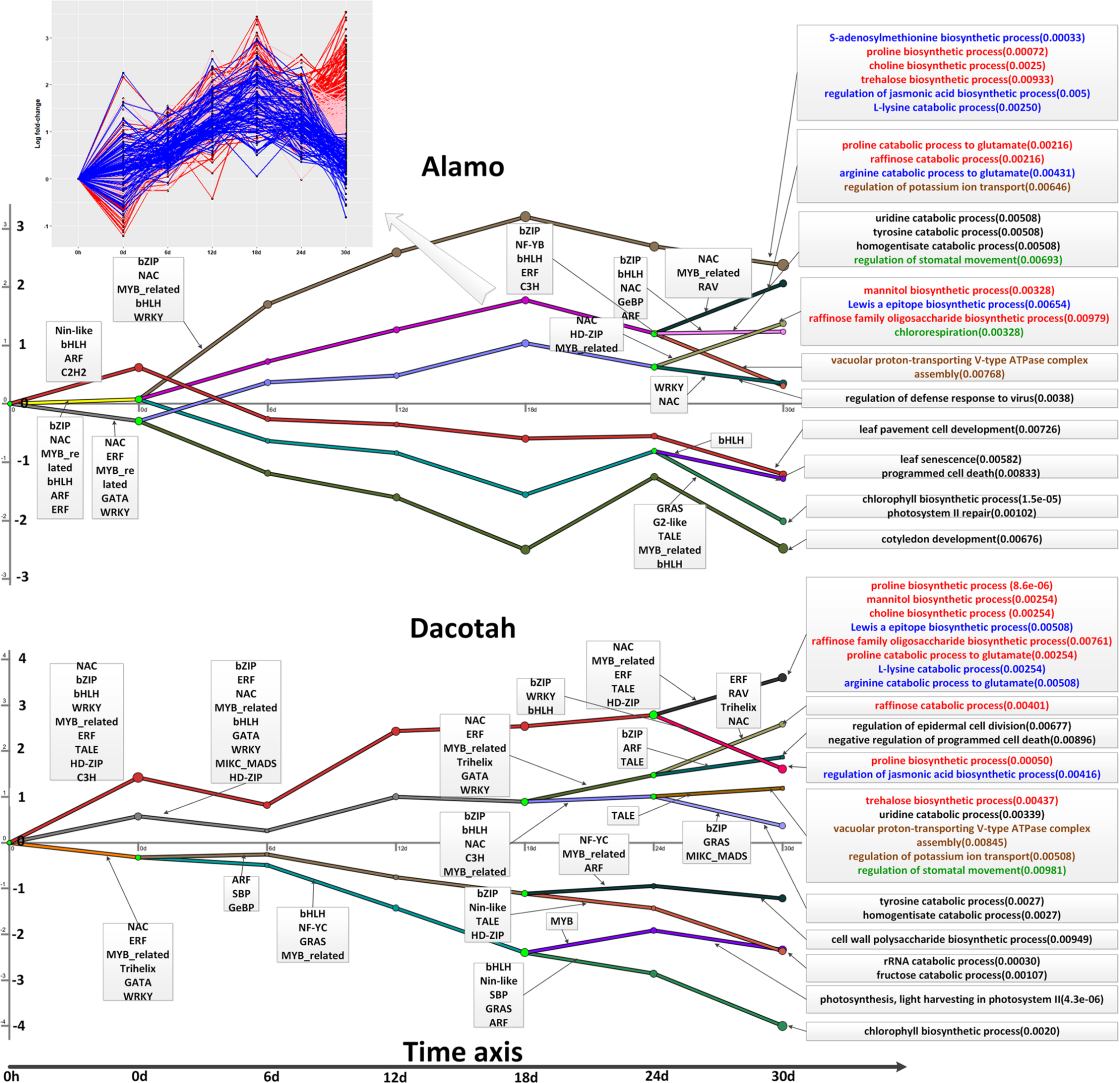
A temporal model for chains of events in each ecotype under drought stress. Each chain represents a collection of co-expressed genes co-regulated by a common set of TFs in the same collection. The y-value of the chain at a specific x point represents the average fold-change (after long transformation) of the expressions of genes represented by the chain at the current point over the expression levels of the same genes at time point 0. Each green node represents a bifurcation event in the network. The TF families (partial) with the highest number of the TFs having split scores below 0.001 appear next to the split that they regulate. The GO functions for each chain are displayed in the right part and the red, green, brown and blue colors indicate different function categories. In each panel, an artificial node is added at 0h so each gene has a “fold change” at time point 0. The panel in the top left corner gives an expanded view of a chain of co-expressed events consisting of one bifurcation event into three sub-chains, being red, pink and blue, respectively.

**Fig 4:**
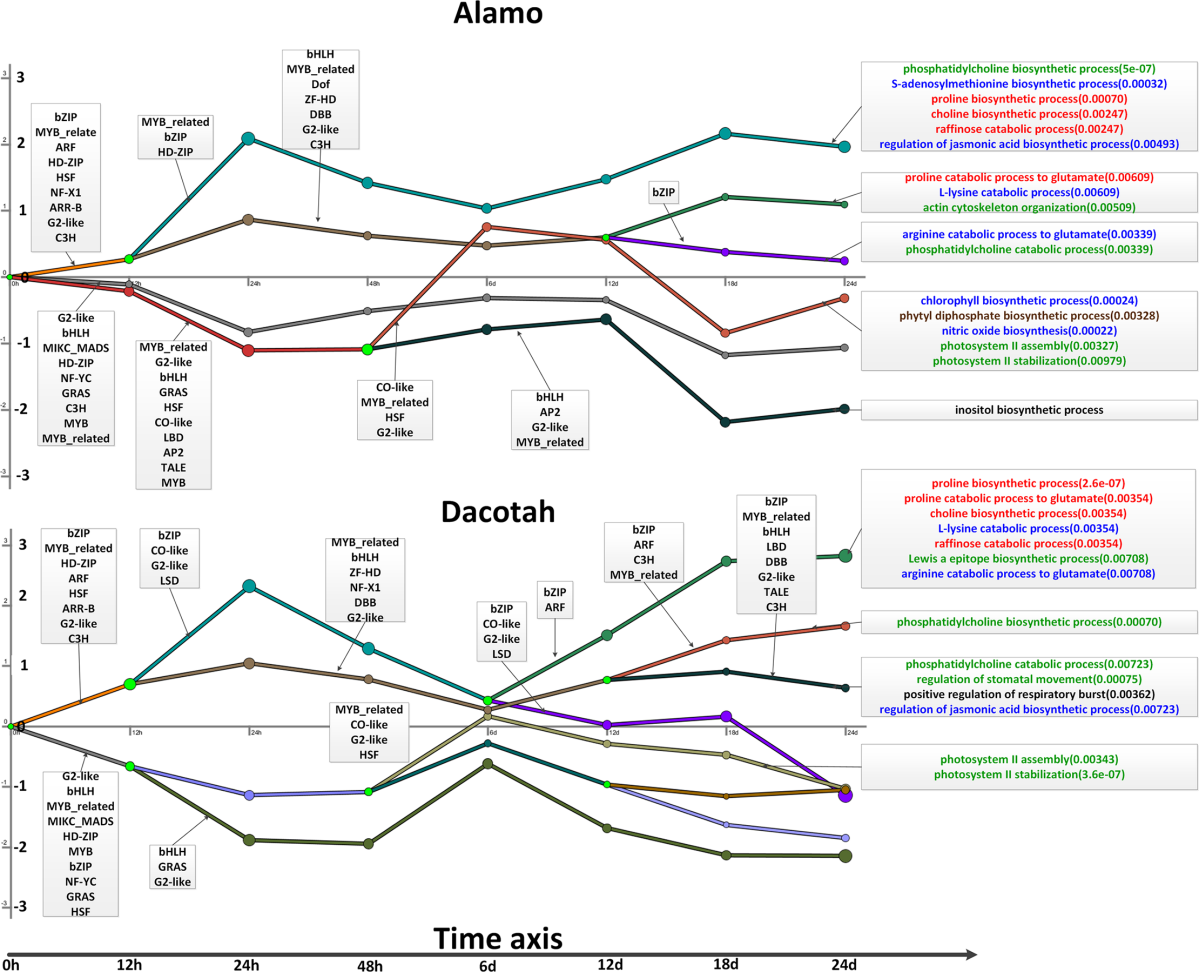
A temporal model for chains of events in each ecotype under salt stress. Each chain represents a collection of co-expressed genes co-regulated by a common set of TFs. The y-value of the chain at a specific x represents the average fold-change (after long transformation) of the expressions of genes represented by the chain at the current point over the expressions of the same genes at time point 0. Each green node represents a bifurcation node. The TF families (partial) with the highest number of TFs with split scores < 0.001 appear next to the split that they regulate. The GO functions for each chain are displayed in the right part and different colors represent different function categories.

Massive responses are observed for each ecotype to each stress type (detailed in S2 and S3 Tables). To put all the response activities into a more logical organization, we have applied the DREM program (see Methods) to the predicted TF-TG interactions and the associated fold-changes of the TGs to construct a temporal model for how the TFs regulate the observed responses, referred to as *regulatory events*, transiting from one time point to the next, represented as an input–output hidden Markov model (IOHMM) [60], which consists of multiple chains of events, as illustrated in Figs 3 and 4. Each chain from the start (time 0) to end (the last time point) represents a collection of co-expressed genes, co-regulated by a common set of TFs. A key property of the model is that it allows the formation of bifurcation events, where a (partial) chain from the start to the current time point can split into two when the set of co-expressed genes up till this time point is split into two subsets of co-expressed genes from this time point on, while the two subsets are not co-expressed beyond the current time point. This process may continue to give rise to multiple bifurcation points. Then, the detailed functions of co-expressed genes for each chain of each ecotype under each stress type are described in S6 File. Overall, the similarities and differences of response activities between two ecotypes responding to each stress fall into the following function categories.

The following summarizes our main observations about how each ecotype responds to drought (Fig 3 and S2 Table) and how these responses may influence various metabolisms [11]:

1. TFs in the families of bZIP, NAC, MYB_related, bHLH and WRKY, which may play roles in the biosynthesis of osmoprotectants such as proline, choline, trehalose, mannitol, glutamate and raffinose-family oligosaccharide, to keep membrane stability under osmotic stress [61–63], are up-regulated in both ecotypes. The main difference between the two ecotypes is: there seems to be more accumulation of the osmoprotectants (like trehalose) in Alamo than in Dacotah, based on the higher expression levels of the relevant biosynthesis genes in Alamo than those in Dacotah, which is consistent with the previous switchgrass metabolic studies under drought stress [11], hence suggesting that the increased levels of these metabolites play a role in Alamo’s drought tolerance, as shown in Fig 3, marked in red;
2. Alamo uses a different mechanism to adapt to drought through utilizing chloro-respiration [64, 65] to prevent damages to the photosynthetic apparatus, which seems to be one of the mechanisms for Alamo to maintain higher *P_r_* levels than Dacotah; and both ecotypes seem to use stomatal movement to avoid excessive water loss through transpiration [66], but fold-changes of relevant genes are slightly different, which may be one reason for why Alamo has higher RWC than Dacotah, as shown in Fig 3, marked in green;
3. Both Alamo and Dacotah are predicted use the following processes to respond to drought: biosynthesis of jasmonic acid [67, 68], S-adenosylmethionine [69], a precursor for the biosynthesis of polyamines known to be involved in response to abiotic stresses [70], which has shown that Almao has higher levels of spermine (one of polyamine) than Dacotah, and conversion of arginine to glutamate [71]; but fold-changes of the relevant genes are significantly different between the two ecotypes, as shown in Fig 3, marked in blue; and
4. Alamo and Dacotah seem to use similar mechanisms to maintain their sodium-ion homeostasis, particularly at the early stage (0d-24d), and potassium transport for maintaining high K^+^/Na^+^ ratios and proton-ATPase for sequestering Na^+^ into the vacuole [14, 72], where a previous study has shown transgenic switchgrass with overexpressed proton-ATPase can enhance growth and salinity tolerance than the wild-type [73]. But the expression levels of the relevant genes are substantially higher in Alamo than in Dacotah. And at a later stage (30d), Alamo expresses higher levels of the potassium-transporter genes than the proton-ATPase biosynthesis genes while Dacotah has these genes expressed with the same fold-changes for the two processes, as shown in Fig 3, marked in brown.

The following summarizes our key observations about how each ecotype may respond to the salt stress (Fig 4, S3 Table):

1. Higher levels of accumulation of osmoprotectants such as proline are predicted in Alamo than in Dacotah, estimated based on the fold changes of genes involved in biosynthesis vs. degradation in the two ecotypes, as shown in Fig 4, marked red;
2. Both Alamo and Dacotah are predicted to have employed the same mechanism to adapt to salt through synthesis of phosphatidylcholine (PC), the major phospholipid in eukaryotic cell membranes known to play a function in the adaptation to salt-mediated hyperosmotic stress [74]. Again more accumulations of PC are predicted in Alamo than in Dacotah based on expression comparisons of genes involved in biosynthesis vs. degradation of the PC and higher expression levels of genes in the assembly and stabilization of photosystem II [53], as shown in Fig 4, marked in green;
3. Alamo seems to have stronger salt tolerance than Dacotah, based on our observation that the former has higher expression levels of genes involved in nitric oxide biosynthesis [72, 75] and the biosynthesis of chlorophyll (Chl), which is consistent with a previous study that Alamo has higher Chl content than Dacotah under salt stress [10], as shown in Fig 4, marked in blue, increases in whose cellular concentrations are known indicators of salt tolerance in crops [76]; and
4. Alamo is predicted to use a different mechanism to reduce oxidative stress through biosynthesis of phytyl diphosphate in the medium stage (6-12d), the prenyl donor for tocopherol biosynthesis [77], as detailed in Fig 4, marked in brown.

Overall, Alamo uses better regulations to respond and adapt to the two stress types than Dacotah.

## Discussion

### Network-based analyses of stress response over time

Plant stress responses, particularly to drought and salt, are dynamic and involve complex cross-talks among multiple metabolic processes and regulations, affecting many aspects of a cellular system, such as osmatic pressure, oxidative stress, ion homeostasis and homeostasis of water supply and demand [78]. Hence, thousands of genes can be differentially expressed once a plant is under drought or salt stress. Traditional gene-centric approaches, such as simple pathway-enrichment analysis, can provide useful information regarding which pathways are differentially expressed, the relationships among such pathways could be very complex to sort out [59]. In this study, we have used a network-based method, focused on key TFs and metabolic TGs. Through organizing the observed events, reflected by coordinated gene-expression changes, as time-dependent Markov models, we have derived a system-level new understanding about how different metabolic processes are linked to a stress and with each other when responding and adapting to a stress. By combining the regulatory networks with the temporal Markov model, we have not only predicted novel information about which transcription regulators are possibly responsible for which parts of the metabolic reprogramming when the plant is stressed, but also gained new understanding about the relative order in executing these reprogrammed metabolisms.

From the detailed analyses of the time-dependent chain events, we can see that (1) similarities as well as differences in terms of when specific metabolic responses, suggested by gene-expression data, to the same stress type take place in the two ecotypes, such as proline or choline taking place approximately at the same time while photosystem II assembly and stabilization happening at different times (Dacotah at 6d and Alamo at 6d-12d, but higher expression levels in Alamo) in the two ecotypes responding to salt; (2) similarities as well as differences between the durations of specific metabolic responses, again reflected by gene-expression data, in the two ecotypes, such as mannitol or trehalose biosynthesis and chloro-respiration responding to drought; (3) responding to salt stress, there is one special time point 6d, a larger decrease in proline and choline biosynthesis in Dacotah than Alamo, a larger increase in the expressions of genes in Chl and phytyl diphosphate biosynthesis in Alamo rather than Dacotah.

### Future work

In the future, we see a few ways to extend our work. One is to design a unified mathematic model to capture all the key time-dependent regulatory and metabolic roles by all genes found to be differentially expressed, which can answer questions through automated analyses of the models such as: (1) what the main and detailed differences between the regulatory systems in response to salt or drought in the two ecotypes are; and (2) what the shared parts between the response systems to drought and salt stresses are. Another area that we plan to do is to enhance our validation procedures for the predicted TF-TG relationships by using more general and sophisticated tools including ChIP-seq data from related plants such as Arabidopsis [20] to compare our predictions against.

## Conclusions

Regulatory networks responding to drought and salt stresses are predicted for two ecotypes of switchgrass, Alamo and Dacotah, based on publicly available gene-expression data of the two ecotypes treated with drought and salt stresses. 40+% of our predicted regulatory relationships are validated against independent data sources. By taking advantage of the time-dependent expression data, we have also constructed time-dependent hidden Markov models to connect the key observed biological activities as reflected by gene-expression data into multiple chains of time-specific events, hence providing a dynamic picture of each of the stress-response processes under study.

## Supporting information

S1 Fig A summary of the differentially expressed TFs for each ecotype under each stress type. (a) Overlapping statistics of differentially expressed TFs. (b) The distribution of TF families for differentially expressed TFs. Note: “Alamo_salt” and “Dacotah_salt” represents the ecotype Alamo and Dacotah under salt stress, respectively; “Alamo_drought” and “Dacotah_drought” represents the ecotype Alamo and Dacotah under drought stress, respectively; and “common” represents the intersections of both ecotypes under both stress types.
(DOCX)

S2 Fig A heat-map of predicted TFA profiles for 143 drought-responding TFs. Hierarchical clustering of the 143 TFs performed using PCC similarities with average linkage method. Rows are for TFs, and columns for samples. Higher and lower levels of activities are indicated with yellow and blue color, respectively.
(DOCX)

S3 Fig A heat-map of predicted TFAs of 110 salt-responding TFs. Hierarchical clustering of the 110 TFs performed using PCC similarities with average linkage method. Rows are for TFs, and columns for samples. Higher and lower levels of activities are indicated with yellow and blue color, respectively.
(DOCX)

S4 Fig Distributions of correlation coefficients for TF-TG pairs predicted by the NCA algorithm, for drought and salt responding networks, respectively. (DOCX)

S5 Fig An inferred GRN for ten TFs with the highest numbers of TGs under salt stress. TFs and TGs are represented as squares and circles, respectively. Genes regarded as stress affected DEGs for both ecotypes, only in Alamo and Dacotah are marked in pink, red and blue, respectively.
(DOCX)

S6 Fig Pair-wise function similarity of co-regulated genes for each TF. (a) Cutoff of gene-pairs similarity based on saturation analysis. (b) Comparison the GO term annotation similarities of pair-wise co-regulated genes for each TF, for drought and salt network, and the corresponding random network, respectively. Note: the pair-wise function similarities for drought and salt network are significantly higher than random generate network, with both p-value less than 2.2*e*^−16^, determined by Wilcoxon rank-sum test.
(DOCX)

S1 Table A summary of samples used in this study. (DOCX)

S2 Table A summary of key up-regulated biological processes for each ecotype responding to drought stress. (DOCX)

S3 Table A summary of key up-regulated biological processes for each ecotype responding to salt stress. (DOCX)

S1 File The DEGs for each ecotype at each time point for each stress. (XLSX)

S2 File The differentially expressed TFs in each ecotype under each stress type. (XLSX)

S3 File The 11,784 and 8,411 predicted TF-TG interactions for drought and salt regulated networks. (XLSX)

S4 File The 503 and 408 validated TF-TG interactions by three related species for drought and salt networks. (XLSX)

S5 File The 5,212 and 3,425 validated TF-TG interactions based on functional relevance of co-regulated genes, for drought and salt networks. (XLSX)

S6 File The TFs, genes and its corresponding GO functions for each chain of each ecotype under each stress type. (XLSX)
