# Supplementary figures and images for: Elucidation and analyses of the regulatory networks of upland and lowland ecotypes of switchgrass in response to drought and salt stresses
Source: PLoS One. 2018 Sep 24;13(9):e0204426. doi: 10.1371/journal.pone.0204426 (PMC6152977; doi:10.1371/journal.pone.0204426)

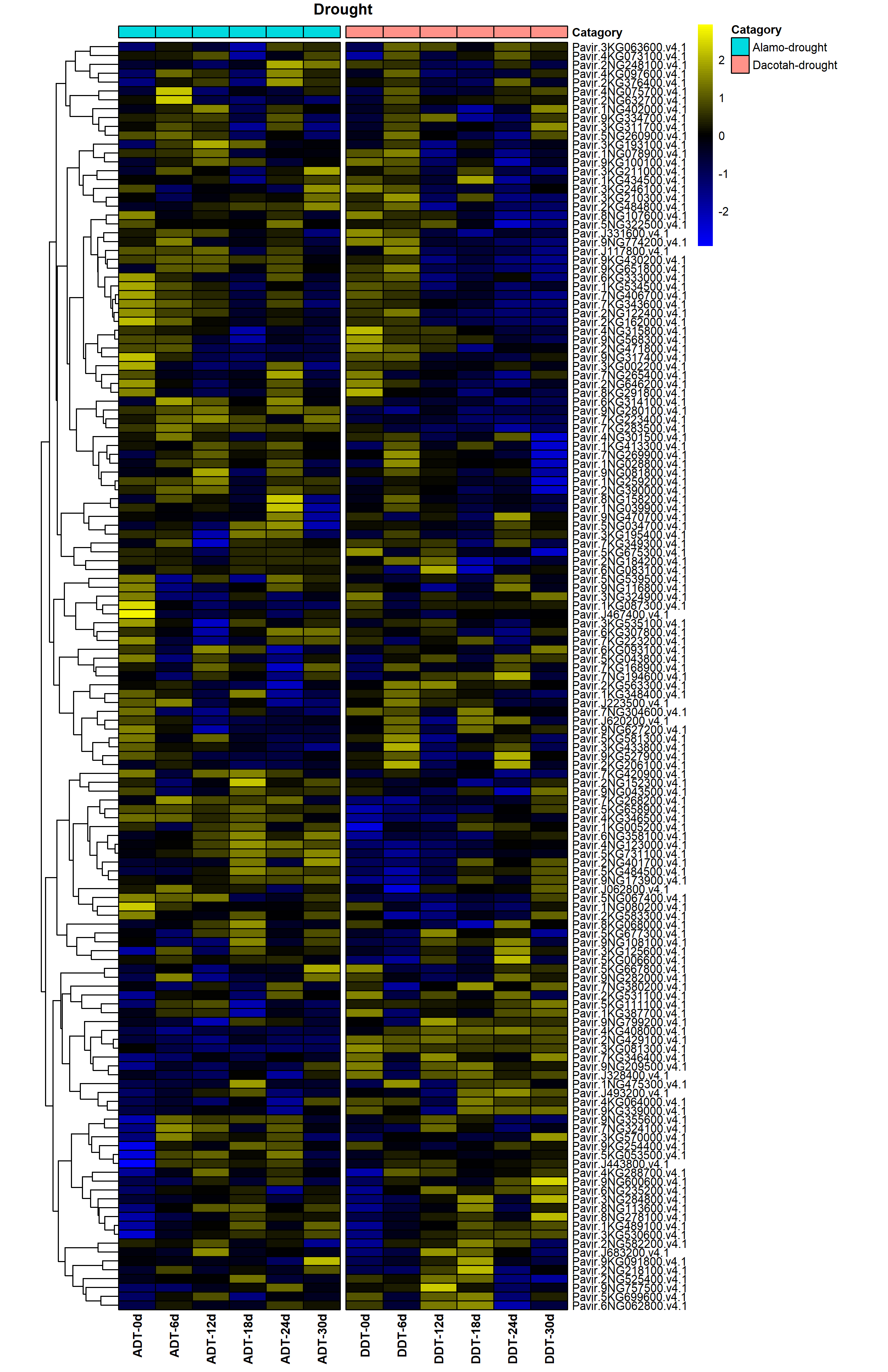

Supplement: S2 Fig — Hierarchical clustering of the 143 TFs performed using PCC similarities with average linkage method. Rows are for TFs, and columns for samples. Higher and lower levels of activities are indicated with yellow and blue color, respectively. (DOCX) [file pone.0204426.s002.docx]

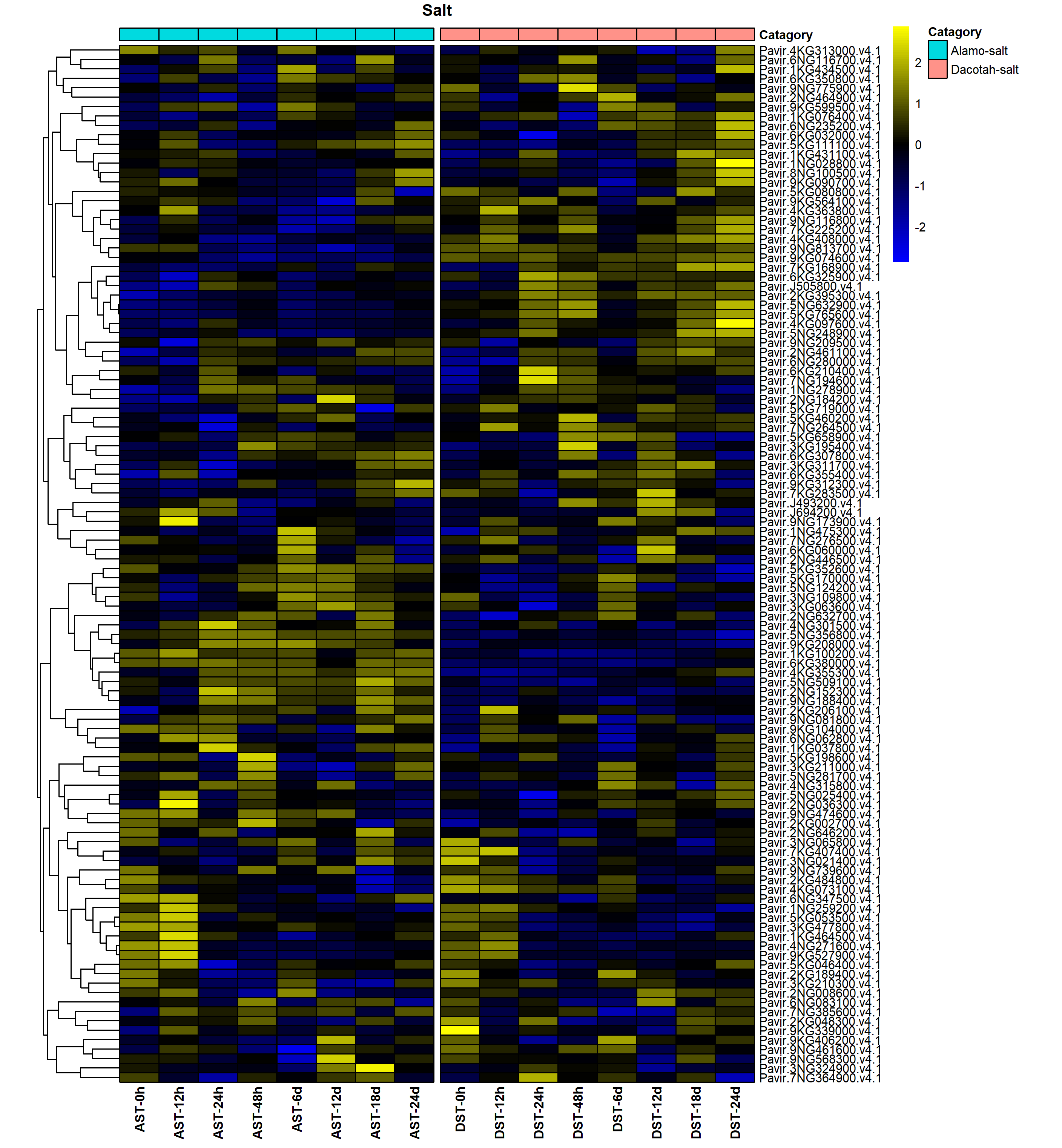

Supplement: S3 Fig — Hierarchical clustering of the 110 TFs performed using PCC similarities with average linkage method. Rows are for TFs, and columns for samples. Higher and lower levels of activities are indicated with yellow and blue color, respectively. (DOCX) [file pone.0204426.s003.docx]

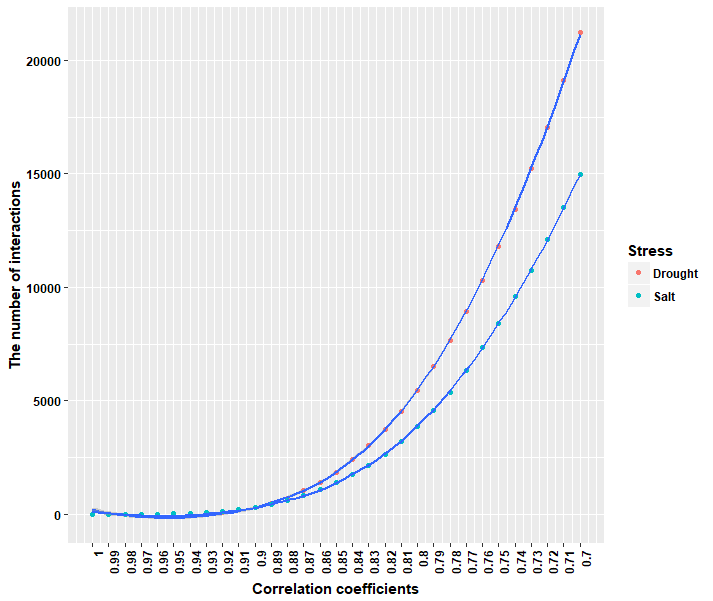

Supplement: S4 Fig — (DOCX) [file pone.0204426.s004.docx]

**
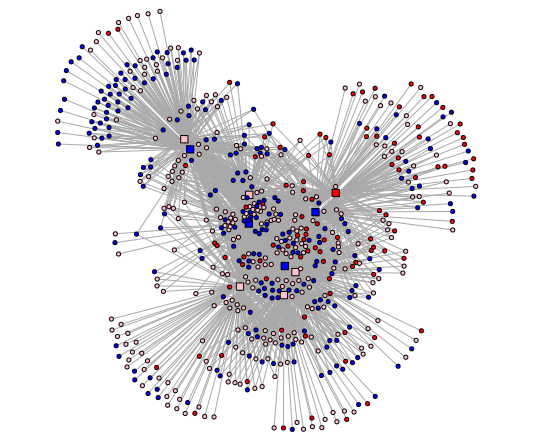
**

Supplement: S5 Fig — TFs and TGs are represented as squares and circles, respectively. Genes regarded as stress affected DEGs for both ecotypes, only in Alamo and Dacotah are marked in pink, red and blue, respectively. (DOCX) [file pone.0204426.s005.docx]
