# Supplementary material for: Elucidation and analyses of the regulatory networks of upland and lowland ecotypes of switchgrass in response to drought and salt stresses
Source: PLoS One. 2018 Sep 24;13(9):e0204426. doi: 10.1371/journal.pone.0204426 (PMC6152977; doi:10.1371/journal.pone.0204426)
Supplement: S1 Table — (DOCX) [file pone.0204426.s007.docx]

| **Project** | **Time course** | **Ecotypes** | **Stress** | **#samples** |
| --- | --- | --- | --- | --- |
| SRP076611 | 0h, 12h, 24h, 48h, 6d, 12d, 18d, 24d | Alamo | Salt | 3, 3, 3, 3, 3, 3, 3, 3 |
|  |  |  | Untreated | 3, 2, 3, 3, 2, 3, 3, 1 |
|  |  | Dacotah | Salt | 3, 3, 3, 3, 3, 3, 3, 3 |
|  |  |  | Untreated | 3, 3, 3, 3, 3, 3, 3, 3 |
| SRP076219 | 0d, 6d, 12d, 18d, 24d, 30d | Alamo | Drought | 3, 3, 3, 3, 3, 3 |
|  |  |  | Untreated | 3, 3, 3, 2, 3, 3 |
|  |  | Dacotah | Drought | 3, 3, 3, 3, 3, 2 |
|  |  |  | Untreated | 3, 3, 3, 3, 3, 3 |
