# Supplementary material for: Elucidation and analyses of the regulatory networks of upland and lowland ecotypes of switchgrass in response to drought and salt stresses
Source: PLoS One. 2018 Sep 24;13(9):e0204426. doi: 10.1371/journal.pone.0204426 (PMC6152977; doi:10.1371/journal.pone.0204426)
Supplement: S2 Table — (DOCX) [file pone.0204426.s008.docx]

| **Category** | **Common** | **Difference** | |
| --- | --- | --- | --- |
|  |  | **Alamo** | **Dacotah** |
| **Response** | hyperosmotic salinity response  response to flooding  response to caffeine  response to estradiol  response to mannitol  cellular response to hypoxia  response to leucine  response to histidine  response to sucrose  cellular response to alcohol  response to wounding | cellular response to anoxia  response to jasmonic acid  cellular response to potassium ion starvation | response to desiccation |
| **Biosynthesis** | proline biosynthetic process  coumarin biosynthetic process  hexitol biosynthetic process  acetyl-CoA biosynthetic process from pyruvate  S-adenosylmethionine biosynthetic process  organophosphate biosynthetic process  mannitol biosynthetic process  choline biosynthetic process  regulation of jasmonic acid biosynthetic process  glycogen biosynthetic process  trehalose biosynthetic process  raffinose family oligosaccharide biosynthetic process |  | Lewis a epitope biosynthetic process |
| **Catabolic** | ornithine catabolic process  oxalate catabolic process  proline catabolic process to glutamate  raffinose catabolic process  L-lysine catabolic process  uridine catabolic process  tyrosine catabolic process  arginine catabolic process to glutamate  homogentisate catabolic process  chlorophyll catabolic process  uracil catabolic process |  |  |
| **Others** | regulation of stomatal movement  regulation of potassium ion transport  proton transport  anatomical structure maturation  cellular iron ion homeostasis | Chlororespiration  seed coat development | regulation of epidermal cell division |
