# Supplementary material for: Elucidation and analyses of the regulatory networks of upland and lowland ecotypes of switchgrass in response to drought and salt stresses
Source: PLoS One. 2018 Sep 24;13(9):e0204426. doi: 10.1371/journal.pone.0204426 (PMC6152977; doi:10.1371/journal.pone.0204426)
Supplement: S3 Table — (DOCX) [file pone.0204426.s009.docx]

| **Category** | **Common** | **Difference** | |
| --- | --- | --- | --- |
|  |  | **Alamo** | **Dacotah** |
| **Response** | response to hexose  hyperosmotic salinity response  response to chlorate  cellular response to selenium ion  cellular response to anoxia  response to glucose | response to alcohol  response to abscisic acid | response to desiccation  response to indolebutyric acid |
| **Biosynthesis** | phosphatidylcholine biosynthetic process  glycogen biosynthetic process  acetyl-CoA biosynthetic process from pyruvate  S-adenosylmethionine biosynthetic process  proline biosynthetic process  choline biosynthetic process  flavonol biosynthetic process  UDP-rhamnose biosynthetic process | phytyl diphosphate biosynthetic process  chlorophyll biosynthetic process  amylopectin biosynthetic process | Lewis a epitope biosynthetic process  raffinose family oligosaccharide biosynthetic process |
| **Catabolic** | proline catabolic process to glutamate  L-lysine catabolic process  raffinose catabolic process  phosphatidylcholine catabolic process |  | uracil catabolic process  chlorophyll catabolic process  ornithine catabolic process  leucine catabolic process  arginine catabolic process to glutamate  inosine catabolic process  triglyceride catabolic process |
| **Others** | regulation of jasmonic acid biosynthetic process  positive regulation of respiratory burst | regulation of stomatal movement  determination of bilateral symmetry  auxin efflux  photosystem II stabilization  photosystem II assembly  nitric oxide mediated signal transduction |  |
