# Supplementary material for: Elucidation and analyses of the regulatory networks of upland and lowland ecotypes of switchgrass in response to drought and salt stresses
Source: PLoS One. 2018 Sep 24;13(9):e0204426. doi: 10.1371/journal.pone.0204426 (PMC6152977; doi:10.1371/journal.pone.0204426)
Supplement: Supplementary file 5 [file pone.0204426.s001.docx]

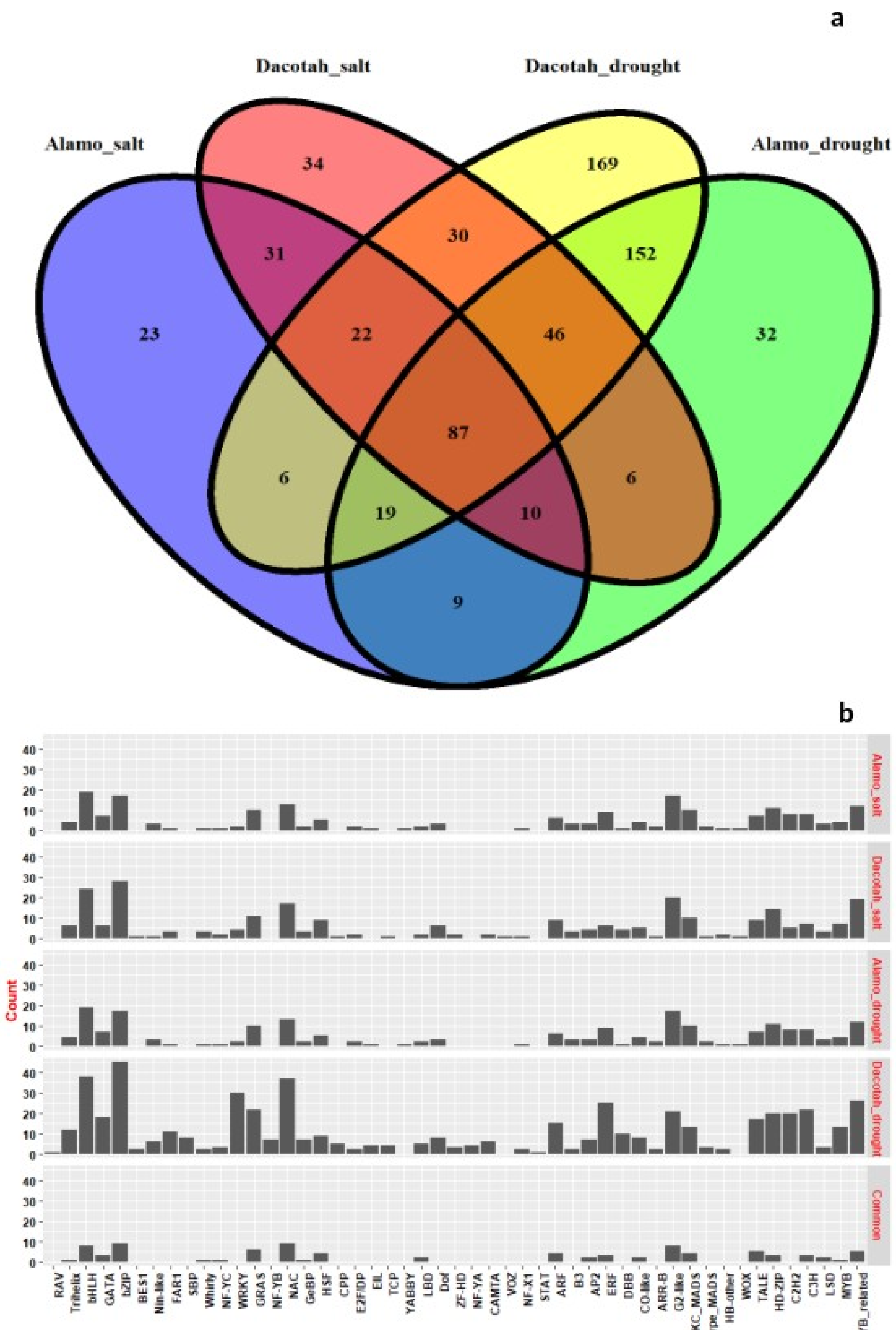
a
Dacotah_salt
Dacotah_drought
Alamo_salt
Alamo_drought
34
169
31
30
152
23
22
46
32
87
6
6
19
10
9
b
Count
Alamo_salt
Dacotah_salt
Alamo_drought
Dacotah_drought
Common
0
10
20
30
40
RAV
Trihelix
bHLH
GATA
bZIP
BES1
Nin-like
FAR1
SBP
Whirly
NF-YC
WRKY
GRAS
NF-YB
NAC
GeBP
HSF
CPP
E2F/DP
EIL
TCP
YABBY
LBD
Dof
ZF-HD
NF-YA
CAMTA
VOZ
NF-X1
STAT
ARF
B3
AP2
ERF
DBB
CO-like
ARR-B
G2-like
MIKC_MADS
M-type_MADS
HB-other
WOX
TALE
HD-ZIP
C2H2
C3H
LSD
MYB
MYB_related
TF family
