# Supplementary material for: Elucidation and analyses of the regulatory networks of upland and lowland ecotypes of switchgrass in response to drought and salt stresses
Source: PLoS One. 2018 Sep 24;13(9):e0204426. doi: 10.1371/journal.pone.0204426 (PMC6152977; doi:10.1371/journal.pone.0204426)
Supplement: Supplementary file 6 [file pone.0204426.s006.docx]

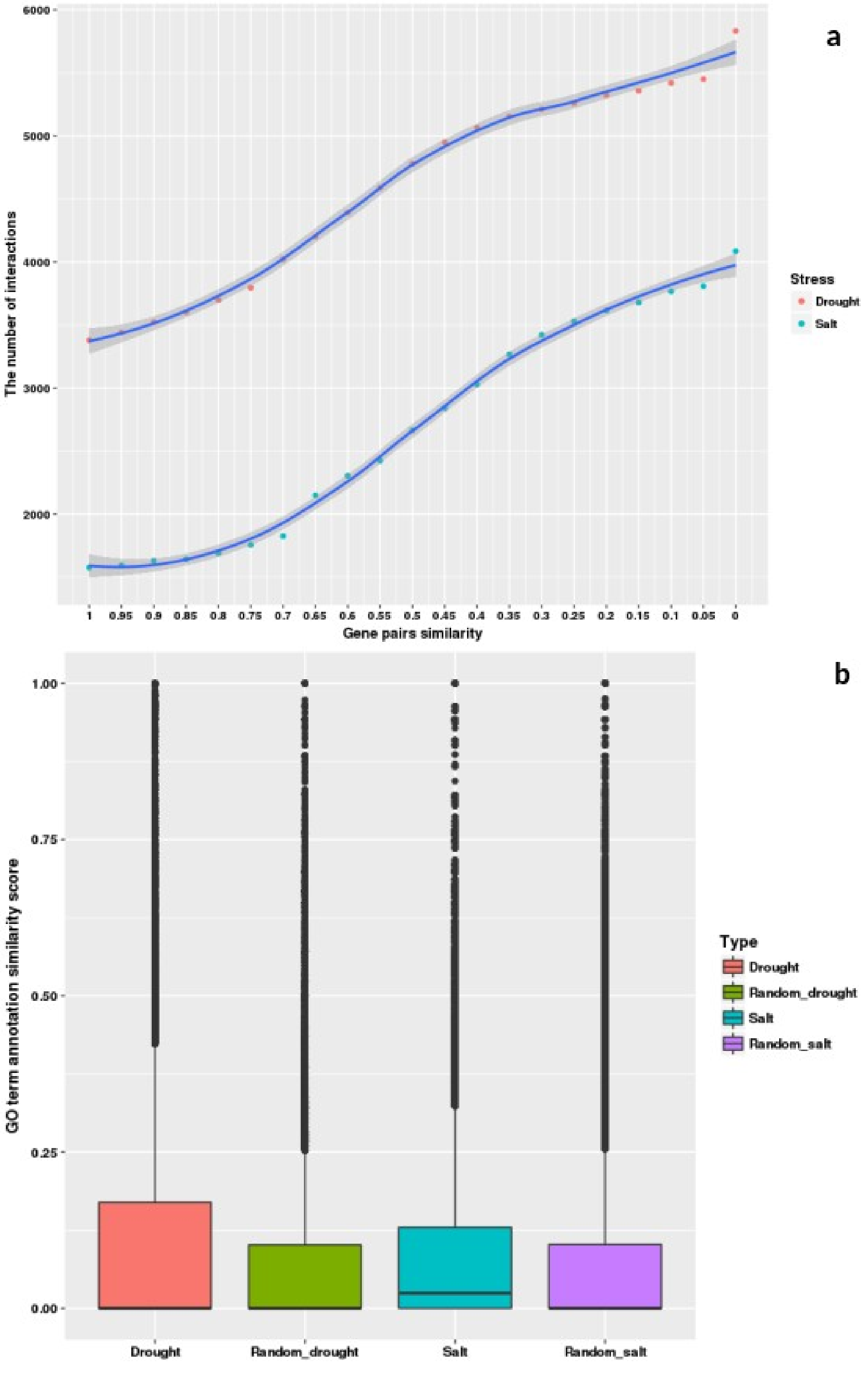

a
6000
5000
4000
3000
2000
The number of interactions
1
0.95
0.9
0.85
0.8
0.75
0.7
0.65
0.6
0.55
0.5
0.45
0.4
0.35
0.3
0.25
0.2
0.15
0.1
0.05
0
Gene pairs similarity
Stress
Drought
Salt
b
1.00
0.75
0.50
0.25
0.00
GO term annotation similarity score
Drought
Random_drought
Salt
Random_salt
Type
Drought
Random_drought
Salt
Random_salt
